# Lamivudine Inhibits *Alu* RNA-induced Retinal Pigment Epithelium Degeneration via Anti-inflammatory and Anti-senescence Activities

**DOI:** 10.1167/tvst.9.8.1

**Published:** 2020-07-01

**Authors:** Kazuhisa Yamada, Hiroki Kaneko, Hideyuki Shimizu, Ayana Suzumura, Rina Namba, Kei Takayama, Seina Ito, Masataka Sugimoto, Hiroko Terasaki

**Affiliations:** 1 Department of Ophthalmology, Nagoya University Graduate School of Medicine, Nagoya, Japan; 2 Department of Ophthalmology, National Defense Medical College, Japan; 3 Department of Mechanism of Aging, National Center for Geriatrics and Gerontology, Obu, Aichi, Japan

**Keywords:** age-related macular degeneration, retinal pigment epithelium, NLRP3 inflammasome, lamivudine, senescence

## Abstract

**Purpose:**

Accumulation of the long noncoding *Alu* element RNA activates the NLRP3 inflammasome and leads to retinal pigment epithelium (RPE) cell death, a key event in the pathogenesis of geographic atrophy during late-stage age-related macular degeneration. Lamivudine (3TC) is a nucleoside analog reverse transcriptase inhibitor known to inhibit the NLRP3 inflammasome. Currently, the intracellular response of the senescence marker p16^Ink4a^ to the long noncoding RNA is being actively studied. The present study aimed to assess the efficacy of 3TC against *Alu* RNA-induced RPE inflammation and senescence by evaluating changes in expression of the proinflammatory cytokines IL-18 and IL-1β and of p16^INK4a^ in RPE cells.

**Methods:**

Cultured human RPE cells and in vivo mouse RPE cells were transfected with an in vitro-transcribed *Alu* RNA, and changes in IL-18, IL-1β, and p16^Ink4a^ expression measured in the presences of 3TC or 3,4-(M)CA as a negative control.

**Results:**

Treatment with 3TC markedly reduced *Alu* RNA-induced expression of IL-18 and IL-1β in human and mouse RPE cells compared with the negative control. Further, *Alu* RNA-induced p16^INK4a^ expression was suppressed by 3TC in human RPE cells.

**Conclusions:**

Our data suggest that *Alu* RNA accumulation contributes to RPE cell senescence in age-related macular degeneration and that this pathogenic process can be suppressed by 3TC.

**Translational Relevance:**

Further verifying this study leads to potential targets for age-related macular degeneration therapy.

## Introduction

*Alu* RNAs are noncoding transcripts that belong to the *Alu* family of retrotransposons and occupy approximately 10% of the human genome as repeat sequences.^1^^–^^4^ *Alu* RNA accumulates in the cells of the retinal pigment epithelium (RPE) of human eyes with geographic atrophy, a part of the late stage of age-related macular degeneration (AMD), owing to deficiency of the RNase DICER1.^5^ Moreover, this intracellular *Alu* RNA accumulation has directly been implicated in the activation of an apoptotic RPE cell death cascade. The nucleotide-binding domain and leucine-rich repeat receptor family member NLRP3 is an intracellular signaling molecule that binds to apoptotic speck-like protein containing a card motif and forms a complex known as the inflammasome.^6^^,^^7^ The NLRP3 inflammasome activates caspase-1, consequently promoting the cleavage of the proinflammatory cytokines IL-18 and IL-1β and yielding their active forms.^8^^–^^13^ Therefore, there is compelling evidence that *Alu* RNA accumulation activates the NLRP3 inflammasome, triggering MyD88 signaling and leading to RPE cell death.^8^

Sterile inflammation, a chronic pathogen-independent low-level inflammatory process, has been implicated in several age-related disorders, including AMD.^8^^,^^14^^,^^15^ IL-18 and IL-1β are central regulators of sterile inflammation^15^; therefore, they are crucial to understand the pathogenesis of AMD. Indeed, a previous study reported that *Alu* RNA stimulation triggered the release of IL-18 (but not IL-1β) from the RPE,^8^ whereas several others have found evidence for inflammasome-mediated IL-1β release.^16^^–^^18^ A recent report demonstrated that nucleoside reverse transcriptase inhibitors inhibit NLRP3 inflammasome activation.^19^ However, whether nucleoside reverse transcriptase inhibitors can inhibit the release of *Alu* RNA-induced IL-18 and IL-1β remains unclear.

The major pathway responsible for *Alu* RNA-evoked inflammasome activation and cell senescence has partially been described. Kerur et al.^20^ presented evidence that cyclic guanosine monophosphate–adenosine monophosphate synthase and stimulator of interferon genes (cGAS/STING) drives interferon signaling and activates the mitochondrial damage-induced NLRP3 inflammasome. Takahashi et al.^21^ reported that activation of the cGAS/STING pathway induces the senescence-associated secretory phenotype and Lan et al.^22^ found that cells from aging disease models displayed STING-dependent expression of the cell cycle regulator p16. Further, De Cecco et al.^23^ reported that LINE-1 retrotransposable elements activate the interferon-1 response and promote age-related inflammation, and Montes et al.^24^ described that the long noncoding RNA MIR31HG regulates p16^INK4a^ expression to moderate senescence. Taken together, these studies indicated the relationship between *Alu* RNA-induced inflammasome activation, enhanced IL-18 and IL-1β cytokine release, and RPE cell senescence via the cGAS/STING pathway.

In the present study, we investigated the effects of *Alu* RNA on IL-18 and IL-1β expression in human cultured and in vivo mouse RPE cells, and examined whether the *Alu* RNA-induced proinflammatory response can be inhibited by the nucleoside reverse transcriptase inhibitor lamivudine (3TC). In addition, we investigated whether *Alu* RNA can induce the senescence marker p16^INK4a^ expression and promote actual RPE degeneration.

## Methods

### In Vitro Transcription of *Alu* RNA

We generated in vitro–transcribed *Alu* RNAs from a linearized T7 promoter-driven *Alu* expression plasmid^5^ with an in vitro transcription kit (AmpriScribe T7-Flash transcription kit, Epicenter, Madison, WI) according to the manufacturer's instructions, and purified the products using a RNA clean up kit (NucleoSpin RNA clean up kit, MACHEREY-NAGEL, Düren, Germany). The integrity of *Alu* RNA was monitored using gel electrophoresis. A 1 µg/µL stock was prepared in water for subsequent transfections. We degraded *Alu* RNA by RNase A (Worthington Biochemical Corporation, Lakewood, NJ) and used it as sham transfection.

### Cell Culture

All cells were maintained at 37°C in 5% CO_2_. Primary human RPE cells (Lonza, Walkersville, MD) were grown in Dulbecco's modified Eagle's medium (Invitrogen, Carlsbad, CA) supplemented with 10% fetal bovine serum (Gibco/ThermoFisher Scientific, Waltham, MA) and 1% penicillin–streptomycin (Merck KGaA, Darmstadt, Germany). Primary human RPE cells at passage 7 were used for all in vitro experiments.

### In Vitro Transfection

Human RPE cells were transfected with 5 nM in vitro-transcribed *Alu* RNA or equimolar degraded *Alu* RNA using Lipofectamine 2000 (Invitrogen) according to the manufacturer's instructions. Cells were pretreated with 100 µM 3TC or the negative control NLRP3 noninhibitor 3,4-(methylenedioxy)cinnamic acid^25^ (3,4-(M)CA, Sigma-Aldrich) 20 minutes before transfection. After 48 hours of transfection, cells were sonicated in radioimmunoprecipitation assay buffer (R0278, Sigma-Aldrich Corp, St. Louis, MO) supplemented with a protease inhibitor cocktail (Roche Diagnostics, Indianapolis, IN) for further enzyme-linked immunosorbent assay (ELISA) studies.

### Mice

The use of animals in the experimental protocol was approved by the Nagoya University Animal Care Committee. All animal experiments were conducted in accordance with the Association for Research in Vision and Ophthalmology Statement for the Use of Animals in Ophthalmic and Vision Research. Male wild-type C57BL/6J mice (CLEA, Tokyo, Japan) from 6 to 8 weeks of age were used in all experiments. Mice were initially anesthetized with an intraperitoneal injection of ketamine and xylazine, and the pupils were dilated with a combination of tropicamide 0.5% and phenylephrine 0.5% (Mydrin-P; Santen, Osaka, Japan) before in vivo RPE cell transfections and 3TC or 3,4-(M)CA treatment.

### In Vivo Transfection and Intravitreal Drug Treatment

*Alu* RNA was transfected into the mouse RPE cells via subretinal injection of 1 µL solution (prepared as described elsewhere in this article) using a 30-gauge needle (Ito Corp., Tokyo, Japan) under a surgical microscope. Before drug treatments, 3TC (FUJIFILM Wako Pure Chemical Corp., Osaka, Japan) was dissolved in phosphate-buffered saline at 125 ng/µL and 3,4-(M)CA^25^ in dimethyl sulfoxide at 105 ng/µL. A 1µL volume of drug solution was intravitreally injected shortly after the subretinal injection of *Alu* RNA. After 1 week, animals were euthanized; the eyes were examined using fundus imaging and were enucleated for further molecular analyses. We isolated mouse RPE cells as previously described.^5^^,^^26^ We washed eyecups with sterile phosphate-buffered saline and created flat mounts. We gently removed the retina to allow RPE layer to be on the surface of the flat mount. We sonicated RPE eyecups in radioimmunoprecipitation assay buffer with supplemented with a protease inhibitor cocktail.

### Fundus Imaging of Mouse Retina

At 7 days after the subretinal injection of 1 µL *Alu* RNA and intravitreal injection of 1 µL 3TC or 3,4-(M)CA, mouse ocular fundus images were acquired using an ultra-widefield imaging system (Optos 200Tx, Optos, Marlborough, MA).

### Zonula Occludens-1 Staining of RPE

To visualize the integrity of the RPE structure, zonula occludens-1 staining was performed as previously described.^5^^,^^8^^,^^27^ Briefly, mouse RPE/choroid flat mounts were fixed with 4% paraformaldehyde and 100% methanol, stained with rabbit antibodies against zonula occludens-1 (1:100; Invitrogen) and visualized with Alexa 594 (Invitrogen). We obtained all images using Nikon confocal microscope (TiE-A1R).

### Enzyme-Linked Immunosorbent Assay

IL-18 and IL-1β expression levels in human RPE cells following the indicated treatments were measured in cell lysates using a human IL-18 ELISA kit (MBL, Nagoya, Japan) and human IL-1β high sensitivity ELISA kit (Invitrogen), respectively, according to the manufacturer's protocols. Similarly, mouse IL-18 and IL-1β expression levels were measured in RPE lysates using a mouse IL-18 ELISA kit (MBL) and mouse IL-1β Quantikine ELISA kit (R&D System, Minneapolis, MN), respectively. Total culture and RPE tissue protein concentrations were determined using a Bradford assay kit (Bio-Rad, Hercules, CA) for normalization of the cytokine expression. Cytokine concentrations were estimated by measuring the absorbance of the ELISA plate at 450 nm (reference at 570 nm) on a microplate reader (Bio-Rad, Richmond, CA).

### Fluorescence Detection of β-Galactosidase–Expressing Human RPE Cells

Human RPE cells were treated with SPiDER-βGal, a stain specific for senescent cells, according to the manufacturer's protocol (Dojin, Kumamoto, Japan). Cells nuclei were counterstained using Hoechst 33342 (Immunochemistry Technologies, Bloomington, MN), after which the cells were imaged using a BioImaging Navigator fluorescence microscope (BZ-9000; Keyence, Osaka, Japan). Fluorescence emission intensity (a measure of senescence) was quantified using NIH ImageJ (http://rsb.info.nih.gov/ij/) and expressed as corrected cellular fluorescence (TCCF = integrated density – [area of selected cell × mean fluorescence of background readings]) according to previous reports.^28^^,^^29^

### Real-Time PCR Measurement of Senescence Marker Expression

Total RNA was isolated using a Qiagen RNeasy Mini Kit (Qiagen Inc., Valencia, CA) and reverse transcribed using the Transcriptor Universal cDNA Master (Roche). The cDNA was amplified using the Thunderbird Probe qPCR mix (Toyobo Life Science, Osaka, Japan) with KOD SYBR qPCR Mix (Toyobo Life Science) and the human p16-INK4a primers F 5’ -GGGGGCACCAGAGGCAGT-3’ and R 5’-GGTTGTGGCGGGGCAGTT-3’. We used human GAPDH F 5’–GGAAGGTGAAGGTCGGAGTCA-3’ and R 5’-GTCATTGATGGCAACAATATCCAC-3’ as housekeeping. The qPCR cycling conditions were 95°C for 10 minutes, followed by 45 cycles of 95°C for 10 seconds, 62°C for 10 seconds, and 72°C for 10 seconds. At the end of amplification, a melting curve analysis was conducted using the dissociation protocol of the sequence detection system to exclude contamination by nonspecific PCR products. Relative expression of the target gene was determined by the 2^−ΔΔCt^ method.

### Statistical Analyses

All data are expressed as mean ± standard error of the mean. Group means from in vitro studies were compared using the Student *t*-test, and the means of in vivo data were compared using the Mann–Whitney *U* test. A *P* value of less than 0.05 was considered significant for all tests.

## Results

### In Vitro Transcription of *Alu* RNA

**Figure 1.**
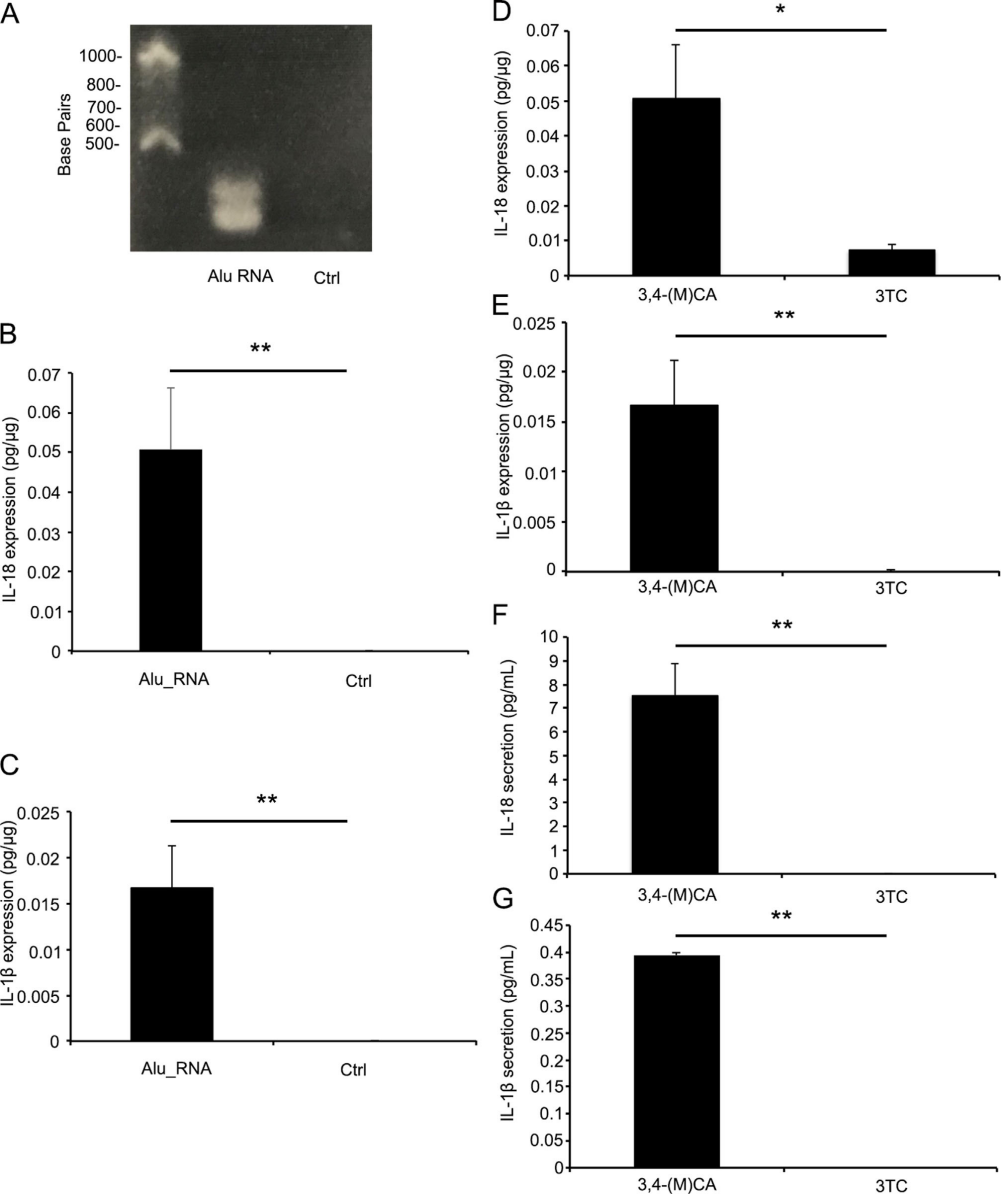
3TC suppresses *Alu* RNA-induced IL-18 and IL-1β expression in human RPE cells. (A) Agarose gel electrophoresis confirming the integrity of in vitro-transcribed *Alu* RNA. Degraded RNA was used as control (Ctrl) and showed no band. (B) IL-18 expression in human RPE cells after 48 hours of *Alu* RNA transfection was higher than Ctrl. (C) IL-1β expression was also higher than Ctrl. (D) Upregulated IL-18 expression in human RPE cells after 48 hours of *Alu* RNA transfection was suppressed by 3TC compared with 3,4-(M)CA. (E) Upregulated IL-1β expression was also suppressed by 3TC compared with 3,4-(M)CA. (F) IL-18 secretion in human RPE cell culture medium after 48 hours of *Alu* RNA transfection was suppressed by 3TC compared with 3,4-(M)CA. (G) IL -1β expression was also suppressed by 3TC compared with 3,4-(M)CA. *P < 0.05. **P < 0.01. *n* = 5 independent human RPE cultures (B–E) *n* = 4 independent human RPE cultures (F, G).

First, we generated in vitro–transcribed *Alu* RNA and verified its integrity by agarose gel electrophoresis. This synthetic *Alu* RNA migrated as a single band with the expected size as previously reported^5^^,^^30^ and degraded *Alu* RNA showed no band (Fig. 1A).

### 3TC Suppressed *Alu* RNA-induced IL-18 and IL-1β Expression in Human RPE Cells

To evaluate the effects of intracellular *Alu* RNA accumulation on IL-18 and IL-1β expression in human RPE cells and potential modulation by 3TC, we measured the post-transcriptional expression of IL-18 and IL-1β in human RPE cell lysate by ELISA following *Alu* RNA or sham transfection and equimolar 3TC or 3,4-(M)CA pretreatment. In *Alu* RNA-transfected human RPE cells, the expression of both cytokines was enhanced compared with sham-transfected controls which were treated with 3,4-(M)CA (Figs. 1B and C), whereas 3TC significantly suppressed IL-18 expression by 86.5 ± 2.9% (*P* = 0.038, Fig. 1D) and IL-1β expression by 98.8 ± 1.0% (*P* = 0.002, Fig. 1E) compared with *Alu* RNA transfection plus 3,4-(M)CA treatment. We also measured the post-transcriptional secretion of IL-18 and IL-1β in human RPE cell culture medium. We could not detect the secretion of both cytokines by 3TC treatment, whereas the secretion of IL-18 and IL-1β was found by 3,4-(M)CA treatment (Figs. 1F and G).

### 3TC Suppressed *Alu* RNA-induced IL-18 and IL-1β Expression in Mouse RPE

**Figure 2.**
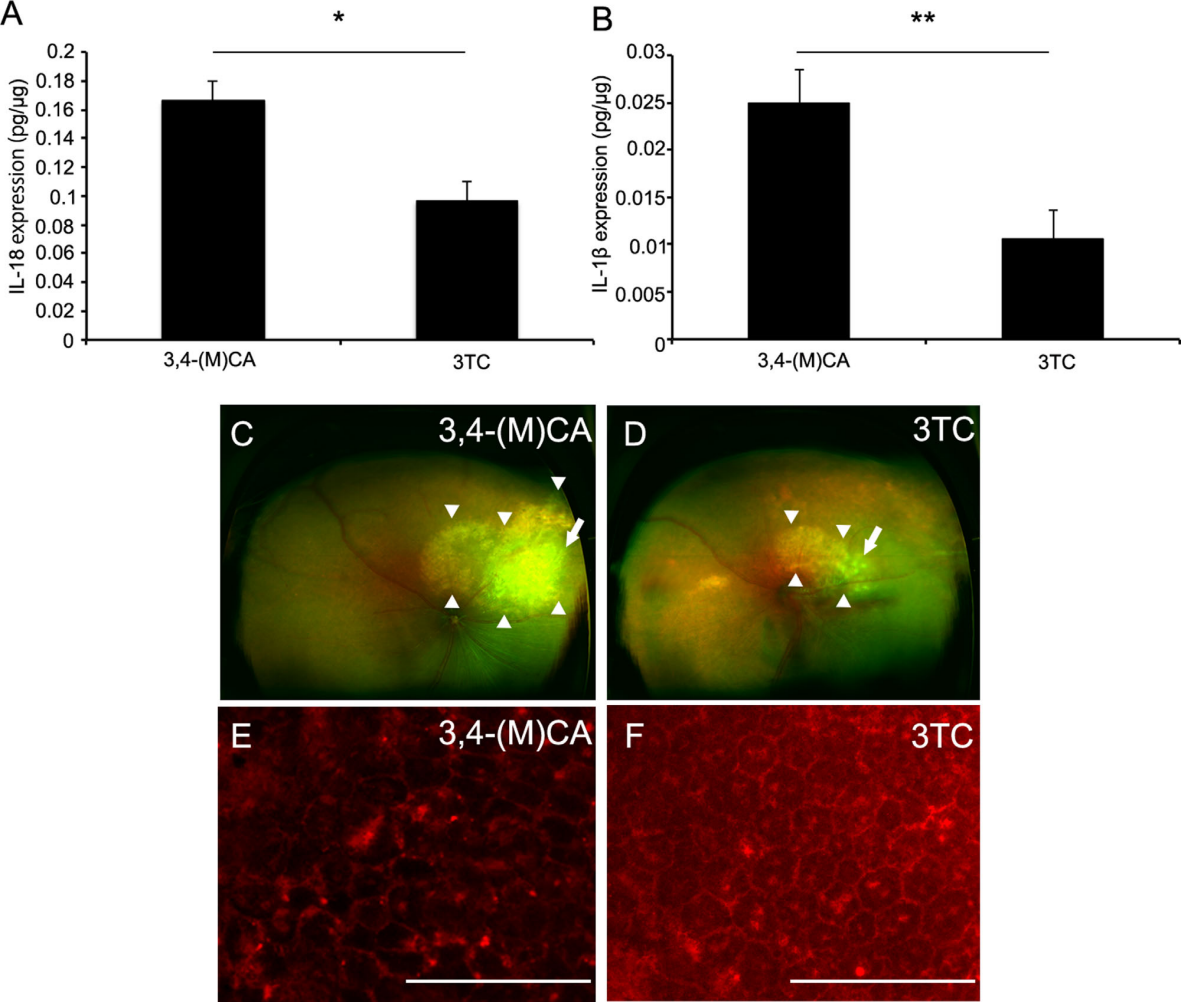
3TC suppresses *Alu* RNA-induced IL-18 and IL-1β expression in mouse RPE. (A) IL-18 expression in mouse RPE at 7 days after subretinal injection of *Alu* RNA was suppressed by 3TC compared with 3,4-(M)CA. (B) IL-1β expression was suppressed by 3TC compared with 3,4-(M)CA. (C, D) Mouse fundus images at 7 days after subretinal injection of *Alu* RNA and intravitreal injection of 3,4-(M)CA (C) or 3TC (D). *Alu* RNA-induced retinal/RPE degeneration was rescued by 3TC compared with 3,4-(M)CA. (E, F) Flat mounts stained for zonula occludens-1 (ZO-1) of mice subretinally injected with *Alu* RNA and Intravitreally injected with 3,4-(M)CA (E) and 3TC (F). ZO-1 image showed damaged RPE structure in the presence of *Alu* RNA was reduced by 3TC compared with 3,4-(M)CA. *P < 0.05. **P < 0.01. *n* = 7–9 (C, D). Arrow: Injection site; arrow head: retinal/RPE degeneration. Scale bars = 100 µm.

Further, we examined the modulatory effects of 3TC on *Alu* RNA-induced IL-18 and IL-1β expression in mouse RPE by a subretinal injection of *Alu* RNA and concomitant intravitreal injection of equimolar 3TC or 3,4-(M)CA. Similar to effects in cultured human RPE cells, 3TC suppressed *Alu* RNA-induced IL-18 expression by 42.1 ± 8.7% (*P* = 0.006) and IL-1β expression by 57.8 ± 12.4% (*P* = 0.030) compared with that observed by 3,4-(M)CA treatment (Figs. 2A and B). Fundus imaging of mouse eye at 7 days after subretinal transfection and intravitreal injection of 3,4-(M)CA revealed substantial retinal/RPE degeneration, whereas intravitreal injection of 3TC substantially reduced *Alu* RNA-induced retinal/RPE degeneration (Figs. 2C and D). The zonula occludens-1 image showed that the disturbed structure of RPE in eyes transfected with *Alu* RNA was reduced by 3TC compared with 3,4-(M)CA (Figs. 2E and F).

### 3TC Suppressed *Alu* RNA-induced Expression of p16^INK4a^

**Figure 3.**
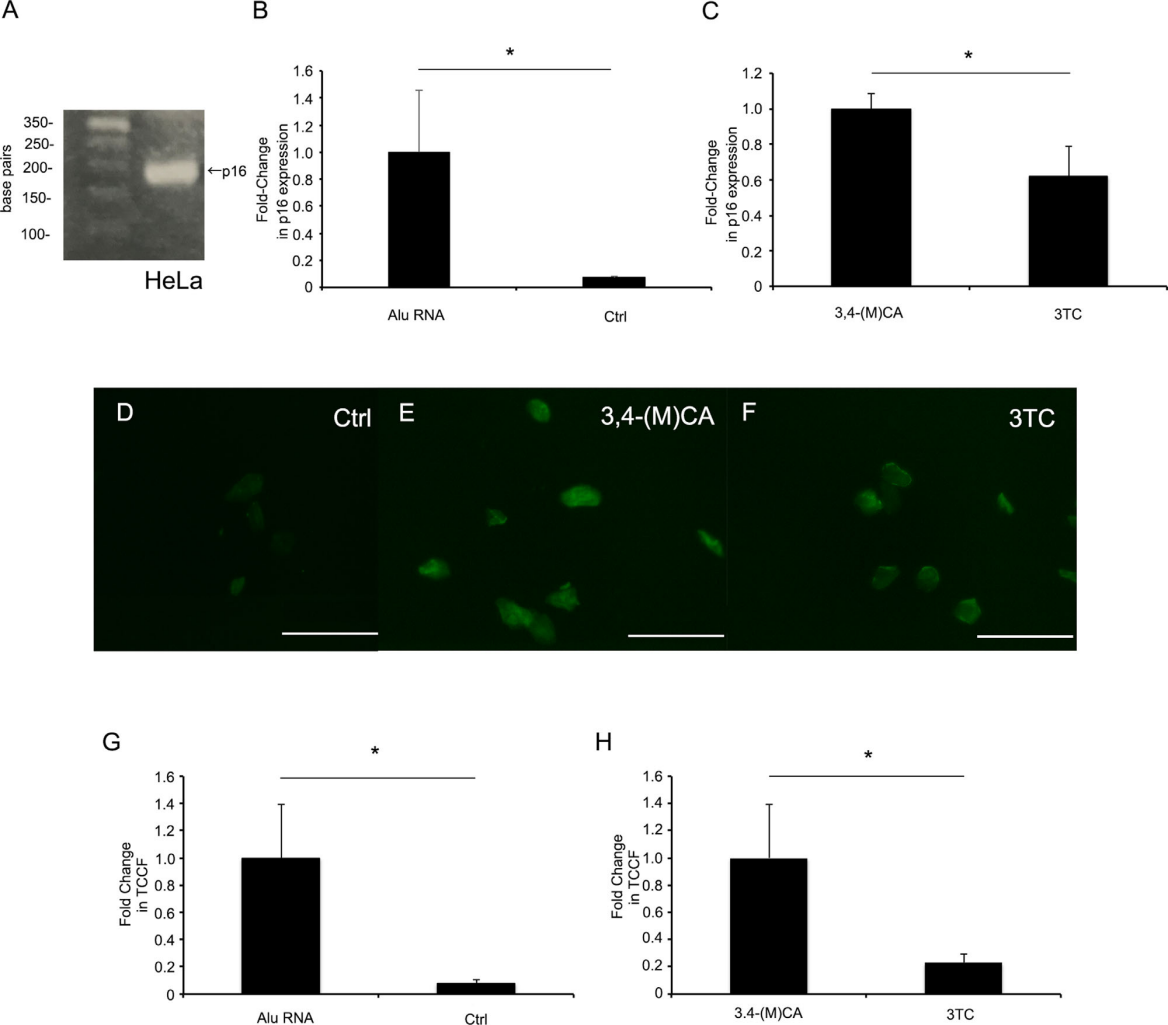
3TC blocks *Alu* RNA-induced senescence of human RPE cells. (A) Agarose gel electrophoresis showing the strong expression of p16^INK4a^ in HeLa cells transfected with *Alu* RNA. (B) p16^INK4a^ expression was upregulated in human RPE cells after 96 hours of *Alu* RNA transfection compared with control (Ctrl). (C) Upregulated p16^INK4a^ expression was suppressed by 3TC compared with 3,4-(M)CA. (D, E, F) *Alu* RNA transfection altered human RPE cell morphology, reduced viability, and enhanced SPiDER-βGal staining intensity (an indicator of cell senescence). (G, H) However, total corrected cellular fluorescence (TCCF) was higher in *Alu* RNA transfected human RPE cells than control and reduced by 3TC compared with 3,4-(M)CA. *P < 0.05. **P < 0.01. *n* = 4 (B, C) *n* = 10 (G, H) Scale bars = 100 µm.

A recent study reported long noncoding RNA-mediated p16^INK4a^ upregulation^23^^,^^24^ and associated inflammation, whereas anti-inflammatory treatment inhibited cell senescence.^31^ Therefore, we examined if *Alu* RNA induces senescence in the RPE and if this response is inhibited by 3TC. To evaluate senescence in human RPE cells, cultures were transfected with 2.5 nM in vitro-transcribed *Alu* RNA for 96 hours using Lipofectamine 2000 and p16^INK4a^ expression was measured using real-time quantitative PCR. To determine the optimal annealing temperature, we used human HeLa cells as a positive control.^32^^,^^33^ After amplification, p16^INK4a^ expression in HeLa cells was detected using agarose gel electrophoresis (Fig. 3A). *Alu* RNA transfected human RPE cells showed increasing expression of p16^INK4a^ compared with control (*P* = 0.039, Fig. 3B). Consistent with anti-senescence efficacy, 3TC decreased p16^INK4a^ expression by 38.2 ± 16.8% compared with 3,4-(M)CA treatment (*P* = 0.005, Fig. 3C).

### Fluorescent Levels of SPiDER-βGal Was Reduced by 3TC

To confirm the pro-senescence effect of *Alu* RNA and the anti-senescence efficacy of 3TC, we stained transfected and drug-treated human RPE cells with SPiDER-βGal. Human RPE cell morphology was altered by *Alu* RNA treatment and viability reduced (Figs. 3D, E, and F) therefore, staining was quantified by the TCCF. *Alu* RNA transfected human RPE showed increasing level of TCCF compared with control (Fig. 3G). Consistent with the anti-senescence effects described in Fig. 2, 3TC reduced TCCF by 77.1% ± 6.7% compared with 3,4-(M)CA (*P* = 0.048, Fig. 3H).

## Discussion

In the present study, we demonstrate that intracellular accumulation of *Alu* RNA in RPE cells can induce the expression of the proinflammatory cytokines IL-18 and IL-1β, upregulate the expression of senescence markers (p16^INK4a^ and SPiDER-βGal staining), and trigger the degeneration of RPE cells. Conversely, these effects were inhibited by 3TC, suggesting that 3TC can rescue the RPE from inflammasome-mediated senescence and degeneration.

IL-1β is an important inflammasome effector. Human drusen extracts induced IL-1β secretion from peripheral blood mononuclear cells stimulated with lipopolysaccharide.^34^ A previous study also suggested that *Alu* RNA can induce IL-18 secretion.^8^ Moreover, it can possibly induce IL-1β secretion via inflammasome activation.^16^^–^^18^ In contrast with previous studies, intracellular *Alu* RNA accumulation upregulated the expression of both IL-18 and IL-1β, possibly owing to the use of in vitro transcribed *Alu* RNA rather than transfection of an *Alu* RNA expression plasmid and the improvement of the sensitivity of IL-1β ELISA kit in recent years. Both IL-18 and IL-1β have potent cytotoxic effects on the RPE.^8^ In contrast, IL-1β has been reported to promote neovascularization, suggesting direct involvement in AMD pathology.^35^ Thus, suppression of IL-1β secretion by 3TC may effectively inhibit neovascularization and RPE degeneration.

Intracellular accumulation of *Alu* RNA promoted senescence of RPE cells as evidenced by upregulated p16^INK4a^ expression and greater fluorescence intensity of SPiDER-βGal staining, and this pro-senescent effect was reversed by 3TC. Recent studies have suggested that inflammation and senescence are strongly related. For instance, the accumulation of *Alu* RNA in endothelial cells induced NLRP3 inflammasome activation and IL-1β production, which in turn promoted p53 and p21 expression, which is its downstream target and an inhibitor of cyclin-dependent kinases that promotes cell cycle arrest (senescence), via the production of reactive oxygen species.^36^ In addition, IL-1β was shown to induce p16^INK4a^ expression in mature chondrocytes.^37^ Our results are consistent with an association between inflammation and senescence as 3TC suppressed *Alu* RNA-induced IL-1β and p16^INK4a^ expression in RPE cells.

Kerur et al.^20^ suggested that *Alu* RNA drives cGAS activation triggered by engagement with mitochondrial DNA. Further, *Alu* RNA-induced NLRP3 inflammasome activation triggers interferon signaling, which can consequently enhance NLRP3 inflammasome expression or activity. STING is an adaptor protein that transduces cGAS-driven interferon signaling.^38^ Lan et al.^22^ reported that knockdown of STING significantly reduced p16^INK4a^ expression in ataxia and progeria cells, and Yang et al.^39^ suggested that cGAS is essential for cellular senescence. Therefore, *Alu* RNA-induced p16^INK4a^ expression may be mediated by the cGAS/STING pathway, and IL-1β may be an important positive modulatory of this process. Further, the accumulation of *Alu* RNA may be an important factor driving sterile inflammation, although further research is required to reveal the mechanism of *Alu* RNA-induced senescence.

At least 72 hours is required for detectable p16^INK4a^ induction in response to environmental stressors.^24^^,^^40^^–^^42^ However, 5 nM of in vitro–transcribed *Alu* RNA significantly damaged human RPE cells, and we were unable to prepare human RPE cell lysate after 72 hours for RT-PCR. Hence, we decreased the *Alu* RNA concentration and extended the transfection period. Concentration and the transfection period may independently influence IL-18, IL-1β, and (or) p16^INK4a^ expression. Nonetheless, we still observed a relationship between inflammation and senescence, underscoring the strength of this association.

We dissolved 3,4-(M)CA in dimethyl sulfoxide and 3TC in phosphate-buffered saline because of the differences of solubility of each protein. The differences in vehicles could affect the results in this study. However, a previous study shows that dimethyl sulfoxide attenuates NLRP3 inflammasome activation.^43^ If we used the same solvent, the result may have been clearer. Thus, we think that the differences in vehicles does not affect the result that 3TC inhibits *Alu* RNA-induced inflammatory and senescence activities. In conclusion, we demonstrated that 3TC can suppress *Alu* RNA-induced IL-18 and IL-1β expression, senescence marker expression, and RPE cell degeneration, further verifying this association and defining potential targets for AMD therapy.
